# Knowledge, attitude, and practice of healthcare workers regarding dengue fever in Mazandaran Province, northern Iran

**DOI:** 10.3389/fpubh.2023.1129056

**Published:** 2023-07-04

**Authors:** Seyed Hassan Nikookar, Mahmood Moosazadeh, Mahmoud Fazeli-Dinan, Morteza Zaim, Mohammad Mehdi Sedaghat, Ahmadali Enayati

**Affiliations:** ^1^Health Sciences Research Center, Department of Medical Entomology and Vector Control, School of Public Health, Mazandaran University of Medical Sciences, Sari, Iran; ^2^Gastrointestinal Cancer Research Center, Non-communicable Diseases Institute, Mazandaran University of Medical Sciences, Sari, Iran; ^3^Department of Medical Entomology and Vector Control, School of Public Health, Tehran University of Medical Sciences, Tehran, Iran; ^4^Department of Medical Entomology and Vector Control, School of Public Health, Mazandaran University of Medical Sciences, Sari, Iran

**Keywords:** knowledge, attitude, practice, dengue, *Aedes*, Mazandaran, northern Iran

## Abstract

**Background:**

Dengue fever is a rapidly emerging infection worldwide with a high public health burden. Adequate training of healthcare workers is essential to warrant the timely provision of health services to improve the outcome of dengue management.

**Methods:**

This is an analytical cross-sectional study, conducted to assess the knowledge, attitudes and practice (KAP) of healthcare workers regarding dengue from April 2021 to March 2022 in Mazandaran Province, northern Iran. Data was collected using a researcher-made structured questionnaire, prepared as Google Forms, and sent to target groups through social media and email. Data analysis was performed by SPSS 22 software using descriptive and inferential statistics (Chi-square) at a significant level of 5%.

**Results:**

Most of the respondents had heard about dengue (83.8%); media (32.7%) and academic education (25%) were the main sources of information. Respondents had less knowledge associated with dengue symptoms (52%) than prevention and control (69%), transmission (72.2%) and clinical management (81%). Based on the 70% cut-off point, the majority of the participants had a good attitude (81%) and practice (73%). However, only 49.6% of the respondents showed good practice regarding dengue local transmission. A significant difference was observed between participants knowledge on clinical management with occupation; attitude with gender and occupation; and practice with gender (*p* < 0.05).

**Conclusion:**

The results of this study revealed gaps in some dimensions of KAP in healthcare workers, therefore, a greater focus should be placed on future training programs to raise knowledge and attitude leading to sound practice and behavior for adequate management of dengue.

## Introduction

Dengue fever (DF) is considered a global public health concern as it is the fastest-growing vector-borne disease over the past five decades (more than 30-fold) in the world (1). The disease is caused by a single-stranded positive-sense RNA virus, a member of the family Flaviviridae and genus Flavivirus (2). There are four distinct immunologically related serotypes of the virus. It is believed that recovery from infection provides lifelong immunity to that serotype, but does not provide cross-protective immunity against each other (3). Although dengue is a self-limiting disease, some patients may progress to severe and life-threatening stages such as dengue hemorrhagic fever or dengue shock syndrome, with a relatively high fatality rate (4). In addition to complications and mortality, dengue can impose a significant economic burden on infected individuals and countries (5), highlighting the importance of the problem.

The first dengue epidemic in Asia, Africa, and North America was recorded between 1779 and 1780. The almost simultaneous outbreak in three continents shows that the virus and its vectors have been distributed around the world in tropical and subtropical regions for more than 200 years. The dengue pandemic began in Southeast Asia after World War II and has since expanded across the world (6). At present, 3.9 billion people are at risk of dengue infection in tropical and subtropical areas (7), with an estimated 390 million cases annually (8), in about 128 countries around the world (7).

Dengue has been an emerging concern in Iran since 2008 when the first imported case was detected in a 58-year-old woman with a history of travel to Malaysia (9). After that, in a retrospective study on suspected cases of Crimean-Congo haemorrhagic fever (CCHF) with haemorrhagic symptoms, 15 probable dengue cases were detected, 8 had a history of travel to Malaysia, India and Thailand, and in 7 cases, the history of travel was not clear, of which 6 cases were from Sistan and Baluchestan Province (10). Reports of imported (11) and suspicious local cases (12) of dengue has been increasing in recent years in Iran. In addition, the presence of dengue vectors, i.e., *Ae. aegypti* and *Ae. albopictus* have been documented in Iran in recent years, highlighting a major concern. In 2008 and 2013, *Ae. albopictus* was reported but not established in Sistan and Baluchestan Province. However, *Ae. aegypti*, the main vector of dengue fever, has been introduced and established in Hormozgan Province, South of Iran (13, 14). To date, no local transmission of the disease is evident in Iran, however, given the establishment of *Ae. aegypti* in southern Iran and the outbreaks/epidemics of dengue in neighboring countries (15, 16), as well as significant travel of Iranians to endemic countries (17), Iran is at risk of dengue epidemics.

Mazandaran ecosystem and weather conditions attract tourists and at the same time provide ideal breeding places for mosquito vectors. There are an international airport and three ports that link the province with Eurasia through the Volga Don Canal (18). In addition, high travel and trade between Mazandaran and Hormozgan Province, where *Ae. aegypti* has newly been established, predispose the province for the entry and spread of *Aedes* vectors and dengue transmission.

Since there is no effective vaccine and specific treatment for the disease, health education and vector control are considered the most important tools for dengue prevention and control. Education of healthcare workers is crucial as they are responsible for prevention (4), control and management of the disease. Capacity is defined as “the ability to carry out stated objectives” (19). “Capacity building” is a systematic process of education, human resources development, individual, collective and organizational knowledge management for the continuous development and improvement of the competencies and capabilities of health personnel, health organization and health system for timely identification, evaluation, selection and application of disease prevention and management protocols (20). For this end, in the past years (before the present study), training workshops and seminars in the form of continuing medical education were held for the healthcare workers in almost all priority provinces regarding dengue fever. The main goal of these workshops was change in the behavior of the healthcare workers to provide adequate services (21). As there are no studies regarding KAP of dengue in Iran, this study is undertaken for the first time to assess the impact of previous health education programs and to plan for further education of healthcare workers regarding the disease and its prevention and control.

## Materials and methods

### Study area

Mazandaran Province is laid in northern Iran between 50°34′–54°10′E and 35°47′–36°35′N, with an area of approximately 23,842 square kilometers and a population of approximately 3,283,582. The province is surrounded by Golestan Province in the East, Guilan Province in the West, Tehran and Semnan Provinces in the South, and the Caspian Sea to the North. Sari is the capital city of the province. The main occupation of the people of the region is agriculture (rice cultivation), horticulture and animal husbandry (cattle, sheep and goats), poultry and fishing. The province is the most popular tourist destination for its natural and historical attractions. There are three active maritime ports and an international airport in the province. The suitable ecosystem and also the points of entry of the province are causes for concern for the entry and spread of the *Aedes* vectors from the northern regions to the country.

### Study design, instruments, and data collection

This is an analytical cross-sectional study, designed in June 2020 after holding workshops and continuing medical education for the health personnel of health centers of the province regarding dengue fever and its vectors. The actual study was conducted to assess KAP in healthcare workers regarding dengue in Mazandaran Province from April 2021 to March 2022 followed by data analysis and synthesis of the results and conclusion in 2023. The individuals who are involved in the diagnosis, prevention, control and management of dengue; and have electronically given consent to take part in the study are eligible to be enrolled in the study population. It includes physicians (n = 853), diseases control staff (n = 186), environmental health engineering (n = 384) and health education (n = 450). The level of education of the respondents was bachelor and master degree for the health experts (i.e., health staff) and general practitioner for the physicians. Considering a 50% knowledge, a 6% margin of error, and a 95% confidence interval, the sample size was calculated 267 according to the sample size formula to estimate proportion or prevalence. However, in practice 284 participants filled in the questionnaires.

The study was designed and planned in two phases, i.e., 1: the questionnaire design and 2: the assessment. In the first phase, a questionnaire was constructed using literature on dengue KAP studies (22) and expert opinions. The questionnaire consisted of four sections: (1) demographic information (gender, workplace, occupation and health information relating to whether the respondent had heard about dengue or not); (2) knowledge of symptoms, transmission routes, clinical management, prevention and control of the disease; (3) attitude towards dengue; (4) preventive measures against dengue, e.g., methods used to reduce breeding places, and potential human-mosquito contact (repellents, bed nets and etc.). The reliability and validity of the questionnaire were evaluated to determine the most appropriate phrases with Cronbach’s alpha coefficient and quantitative-qualitative face and content validity, the results of which was published earlier (23). The English version of the survey instrument is available in appendix 1. In the second phase, the questionnaire was transformed into the Google Forms, and its link was made available to the healthcare workers (health experts and doctors) on the website of the health department. An administration team was devised and followed up the whole process of data collection including sending monthly reminders through social media (WhatsApp) and e-mails to the study participants as well as dealing with the respondents in case any assistance deemed necessary. The study period was coincided with the COVID-19 that caused some limitations in data collection, therefore, available individuals were recruited and enrolled in the study.

### Data analysis

All completed questionnaires were double-checked and confirmed for structural completion and compatibility. KAP assessment was executed using a scoring system. In Knowledge and practice assessment, responses to questions were coded such that correct answers (supported by current literature) were scored 1 and incorrect answers were scored 0. The total score for knowledge and practice was 50 and 16, respectively. Knowledge was assessed based on the questions grouped under the following four categories: Knowledge regarding (1) symptoms (2) transmission (3) clinical management (4) prevention and control. For attitude, a five-point Likert-like scale was applied to answers to the questions, i.e., 1 = strongly disagree; 2 = somewhat disagree; 3 = neither agree nor disagree; 4 = somewhat agree; and 5 = strongly agree. The attitude score was computed as the sum of the participant’s correct responses. A range of cut-offs for the different components of KAP (70–75%) was devised and used by other researches (24), therefore, in this study a cut-off point of 70% was set to differentiate between the groups “poor” and “good” KAP; the respondents were considered to have adequate knowledge, attitude and practice if the score in each was above 70%.

The obtained data were analyzed using descriptive statistics (frequency, mean and standard deviation) and inferential statistics (Chi-square). SPSS software version 22 was used for the analysis value of *p* ≤0.05 was considered statistically significant.

## Results

### Demographic characteristics of healthcare workers

**Figure 1:**
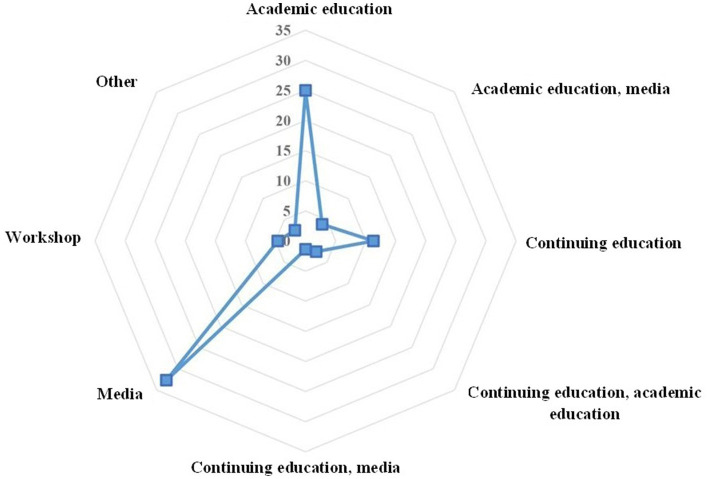
Source of information on dengue fever among healthcare workers in Mazandaran Province, northern Iran, 2022.

A total of 284 participants successfully filled in and returned the questionnaire, of whom 66.2% were female. Most of the study population were health experts (60.9%%) and the rest were physicians (39.1%). The majority worked in the public health sector (87.7%) and stated that they had heard of dengue (83.8%) (Table 1). Regarding the information sources about dengue fever (Figure 1), most participants reported that they heard about dengue fever through media (32.7%) and academic education (25%) followed by continuing education (11.1%), workshops (4%), academic education plus media (3.9%).

**Table 1:** Demographic characteristics of healthcare workers participating in the knowledge, attitude and practice study on dengue disease in Mazandaran Province, northern Iran, 2022.

	Characteristics	*n*	%
Total		284	100
Gender	Male	96	33.8
	Female	188	66.2
Occupation	General physician	111	39.1
	Health experts	173	60.9
Workplace (sector)	Private	35	12.3
	Public	249	87.7
Have you heard of dengue?	Yes	238	83.8
	No	46	16.2

### Knowledge about dengue fever

More than half of the participants (n = 149, 52.5%) were able to correctly identify general symptoms of dengue disease such as fever, headache, joint and muscle pain, pain behind the eyes, rash and abdominal pain. Fifty-three percent of females, 56.8% (*n* = 63) of physicians, 65.7% (n = 23) of those in the private sector responded correctly to the questions regarding the symptoms of dengue.

Seventy-two percent of respondents were aware of the fact that *Aedes* mosquitoes are the main vector of dengue, mostly transmitted during the day by biting *Aedes*. The knowledge regarding transmission was 71.9% (*n* = 179) in public sector, 74.3% (*n* = 26) in private sector, and 77.1% (*n* = 74) in male, 69.7% (*n* = 131) in female, and 69.9% (*n* = 121) in health experts and 75.7% (*n* = 84) in physicians.

Eighty-one percent (*n* = 230) and 69% (*n* = 196) of the respondents knew about clinical management and prevention and control of dengue disease, respectively. There was no significant difference between the respondents’ knowledge about symptoms, transmission, or clinical management of dengue based on workplace demographic variables and gender, while it was statistically significant between physicians and health experts (90.1% vs. 75.1%, respectively, *p* = 0.001) regarding clinical management, and the private and public sectors (85.7% vs. 66.7%, respectively, *p* = 0.015) regarding disease prevention and control (Table 2).

**Table 2:** Knowledge on symptoms, transmission, clinical management and prevention of dengue fever (DF) among healthcare workers in Mazandaran Province, northern Iran, 2022.

Variables	Total		Private sector	Public sector	Sig.	Female	Male	Sig.	Physician	Health experts	Sig.
	Answer	*n* (%)	*n* (%)	*n* (%)		*n* (%)	*n* (%)		*n* (%)	*n* (%)	
Knowledge of symptoms	Correct	149 (52.5)	23 (65.7)	126 (50.6)	0.06	100 (53.2)	49 (51.0)	0.414	63 (56.8)	86 (49.7)	0.150
	Incorrect	135 (47.5)	12 (34.3)	123 (49.4)		88 (46.8)	47 (49.0)		48 (43.2)	87 (50.3)	
Knowledge of transmission	Correct	205 (72.2)	26 (74.3)	179 (71.9)	0.471	131 (69.7)	74 (77.1)	0.119	84 (75.7)	121 (69.9)	0.180
	Incorrect	79 (27.8)	9 (25.7)	70 (28.1)		57 (30.3)	22 (22.9)		27 (24.3)	52 (30.1)	
Knowledge of clinical management	Correct	230 (81.0)	30 (85.7)	200 (80.3)	0.307	155 (82.4)	75 (78.1)	0.235	100 (90.1)	130 (75.1)	0.001
	Incorrect	54 (19.0)	5 (14.3)	49 (19.7)		33 (17.6)	21 (21.9)		11 (9.9)	43 (24.9)	
Knowledge of prevention and control	Correct	196 (69.0)	30 (85.7)	166 (66.7)	0.015	125 (66.5)	71 (74.0)	0.124	76 (68.5)	120 (69.4)	0.487
	Incorrect	88 (31.0)	5 (14.3)	83 (33.3)		63 (33.5)	25 (26.0)		35 (31.5)	53 (30.6)	

### Attitudes about dengue fever

Table 3 shows that the majority of the respondents strongly agreed (47.2%) and agreed (46.8%) that dengue is a dangerous illness with a significant difference between the private and public sectors (*p* = 0.05) and gender (*p* = 0.032). Eighty-seven percent had a positive attitude towards the fact that Iran is at risk of invasive vectors of dengue with a significant difference in gender (*p* = 0.007). The majority of the respondents strongly agreed/agreed (91.9%) that dengue is a preventable disease and source reduction of dengue vectors is a control strategy [strongly agreed (64.4%) and agreed (31.7%)], figures statistically different between health experts and physicians (*p* = 0.039).

**Table 3:** Attitude towards dengue fever (DF) among healthcare workers in Mazandaran Province, northern Iran, 2022.

	Answer	Total	Private sector	Public section	*p* value	Female	Male	*p* value	Physician	Health experts	*p* value
		*n* (%)	*n* (%)	*n* (%)		*n* (%)	*n* (%)		*n* (%)	*n* (%)	
1. In your opinion, dengue is a dangerous disease?	Very disagree	1 (0.4)	0 (0.0)	1 (0.4)	0.050	1 (0.5)	0 (0.0)	0.032	0 (0.0)	1 (0.6)	0.363
	Very agree	134 (47.2)	20 (57.1)	114 (45.8)		77 (41.0)	57 (59.4)		58 (52.3)	76 (43.9)	
	disagree	1 (0.4)	1 (2.9)	0 (0.0)		1 (0.5)	0 (0.0)		1 (0.9)	0 (0.0)	
	not sure	15 (5.3)	2 (5.7)	13 (5.2)		13 (6.9)	2 (2.1)		6 (5.4)	9 (5.2)	
	agree	133 (46.8)	12 (34.3)	121 (48.6)		96 (51.1)	37 (38.5)		46 (41.4)	87 (50.3)	
2-Is Iran at risk of invasive vectors of dengue?	Very disagree	1 (0.4)	0 (0.0)	1 (0.4)	0.866	1 (0.5)	0 (0.0)	0.007	0 (0.0)	1 (0.6)	0.288
	Very agree	92 (32.4)	11 (31.4)	81 (32.5)		53 (28.2)	39 (40.6)		40 (36.0)	52 (30.1)	
	disagree	5 (1.8)	0 (0.0)	5 (2.0)		5 (2.7)	0 (0.0)		2 (1.8)	3 (1.7)	
	not sure	31 (10.9)	3 (8.6)	28 (11.2)		28 (14.9)	3 (3.1)		7 (6.3)	24 (13.9)	
	agree	155 (54.6)	21 (60.0)	134 (53.8)		101 (53.7)	54 (56.2)		62 (55.9)	93 (53.8)	
3-Is the dengue disease preventable?	Very disagree	1 (0.4)	0 (0.0)	1 (0.4)	0.388	1 (0.5)	0 (0.0)	0.064	0 (0.0)	1 (0.6)	0.676
	Very agree	111 (39.1)	9 (25.7)	102 (41.0)		66 (35.1)	45 (46.9)		41 (36.9)	70 (40.5)	
	disagree	1 (0.4)	0 (0.0)	1 (0.4)		1 (0.5)	0 (0.0)		0 (0.0)	1 (0.6)	
	not sure	21 (7.4)	2 (5.7)	19 (7.6)		19 (10.1)	2 (2.1)		7 (6.3)	14 (8.1)	
	agree	150 (52.8)	24 (68.6)	126 (50.6)		101 (53.7)	49 (51.0)		63 (56.8)	87 (50.3)	
4-Is the control against dengue vectors a strategy to prevent the disease?	Very disagree	1 (0.4)	0 (0.0)	1 (0.4)	0.741	1 (0.5)	0 (0.0)	0.365	0 (0.0)	1 (0.6)	0.039
	Very agree	183 (64.4)	25 (71.4)	158 (63.5)		114 (60.6)	69 (71.9)		83 (74.8)	100 (57.8)	
	disagree	2 (0.7)	0 (0.0)	2 (0.8)		1 (0.5)	1 (1.0)		1 (0.9)	1 (0.6)	
	not sure	8 (2.8)	0 (0.0)	8 (3.2)		6 (3.2)	2 (2.1)		1 (0.9)	7 (4.0)	
	agree	90 (31.7)	10 (28.6)	80 (32.1)		66 (35.1)	24 (25.0)		26 (23.4)	64 (37.0)	
5-Do you think that tires, dishes and pots around the house are the proper places for the development of dengue vectors?	Very disagree	1 (0.4)	0 (0.0)	1 (0.4)	0.862	1 (0.5)	0 (0.0)	0.002	0 (0.0)	1 (0.6)	0.613
	Very agree	123 (43.3)	17 (48.6)	106 (42.6)		67 (35.6)	56 (58.3)		51 (45.9)	72 (41.6)	
	disagree	5 (1.8)	0 (0.0)	5 (2.0)		4 (2.1)	1 (1.0)		3 (2.7)	2 (1.2)	
	not sure	46 (16.2)	6 (17.1)	40 (16.1)		39 (20.7)	7 (7.3)		15 (13.5)	31 (17.9)	
	agree	109 (38.4)	12 (34.3)	97 (39.0)		77 (41.0)	32 (33.3)		42 (37.8)	67 (38.7)	
6-Do you think people should actively participate in dengue control?	Very disagree	1 (0.4)	0 (0.0)	1 (0.4)	0.582	1 (0.5)	0 (0.0)	0.131	0 (0.0)	1 (0.6)	0.032
	Very agree	144 (50.7)	20 (57.1)	124 (49.8)		87 (46.3)	57 (59.4)		69 (62.2)	75 (43.4)	
	disagree	1 (0.4)	0 (0.0)	1 (0.4)		0 (0.0)	1 (1.0)		0 (0.0)	1 (0.6)	
	not sure	16 (5.6)	0 (0.0)	16 (6.4)		12 (6.4)	4 (4.2)		4 (3.6)	12 (6.9)	
	agree	122 (43.0)	15 (42.9)	107 (43.0)		88 (46.8)	34 (35.4)		38 (34.2)	84 (48.6)	
7- Do you think just the government is responsible for controlling dengue vectors?	Very disagree	41 (14.4)	7 (20.0)	34 (13.7)	0.082	24 (12.8)	17 (17.7)	0.059	17 (15.3)	24 (13.9)	0.04
	Very agree	27 (9.5)	7 (20.0)	20 (8.0)		12 (6.4)	15 (15.6)		12 (10.8)	15 (8.7)	
	disagree	137 (48.2)	14 (40.0)	123 (49.4)		95 (50.5)	42 (43.8)		55 (49.5)	82 (47.4)	
	not sure	30 (10.6)	1 (2.9)	29 (11.6)		23 (12.2)	7 (7.3)		10 (9.0)	20 (11.6)	
	agree	49 (17.3)	6 (17.1)	43 (17.3)		34 (18.1)	15 (15.6)		17 (15.3)	32 (18.5)	
8-Do you think molecular test is used to confirm dengue?	Very disagree	2 (0.7)	0 (0.0)	2 (0.8)	0.909	1 (0.5)	1 (1.0)	0.342	1 (0.9)	1 (0.6)	0.358
	Very agree	41 (14.4)	6 (17.1)	35 (14.1)		23 (12.2)	18 (18.8)		15 (13.5)	26 (15.0)	
	disagree	26 (9.2)	3 (8.6)	23 (9.2)		15 (8.0)	11 (11.5)		15 (13.5)	11 (6.4)	
	not sure	110 (38.7)	15 (42.9)	95 (38.2)		79 (42.0)	31 (32.3)		40 (36.0)	70 (40.5)	
	agree	105 (37.0)	11 (31.4)	94 (37.8)		70 (37.2)	35 (36.5)		40 (36.0)	65 (37.6)	
9-Do you think ELISA test is used to confirm dengue?	Very disagree	4 (1.4)	1 (2.9)	3 (1.2)	0.353	4 (2.1)	0 (0.0)	0.276	3 (2.7)	1 (0.6)	0.007
	Very agree	43 (15.1)	2 (5.7)	41 (16.5)		27 (14.4)	16 (16.7)		12 (10.8)	31 (17.9)	
	disagree	26 (9.2)	2 (5.7)	24 (9.6)		21 (11.2)	5 (5.2)		14 (12.6)	12 (6.9)	
	not sure	107 (37.7)	14 (40.0)	93 (37.3)		70 (37.2)	37 (38.5)		32 (28.8)	75 (43.4)	
	agree	104 (36.6)	16 (45.7)	88 (35.3)		66 (35.1)	38 (39.6)		50 (45.0)	54 (31.2)	
10-Do you think the report of dengue is a national priority?	Very disagree	1 (0.4)	0 (0.0)	1 (0.4)	0.834	0 (0.0)	1 (1.0)	0.135	1 (0.9)	0 (0.0)	0.012
	Very agree	97 (34.2)	14 (40.0)	83 (33.3)		59 (31.4)	38 (39.6)		49 (44.1)	48 (27.7)	
	disagree	6 (2.1)	0 (0.0)	6 (2.4)		3 (1.6)	3 (3.1)		0 (0.0)	6 (3.5)	
	not sure	35 (12.3)	4 (11.4)	31 (12.4)		28 (14.9)	7 (7.3)		11 (9.9)	24 (13.9)	
	agree	145 (51.1)	17 (48.6)	12 (51.4)		98 (52.1)	47 (49.0)		50 (45.0)	95 (54.9)	
11-Do you think that dengue disease can be treated?	Very disagree	4 (1.4)	0 (0.0)	4 (1.6)	0.590	4 (2.1)	0 (0.0)	0.003	1 (0.9)	3 (1.7)	0.172
	Very agree	56 (19.7)	5 (14.3)	51 (20.5)		30 (16.0)	26 (27.1)		20 (18.0)	36 (20.8)	
	disagree	7 (2.5)	1 (2.9)	6 (2.4)		3 (1.6)	4 (4.2)		0 (0.0)	7 (4.0)	
	not sure	56 (19.7)	10 (28.6)	46 (18.5)		31 (16.5)	25 (26.0)		26 (23.4)	30 (17.3)	
	agree	161 (56.7)	19 (54.3)	142 (57.0)		120 (63.8)	41 (42.7)		64 (57.5)	97 (56.1)	
12-Is the follow-up of suspected dengue disease necessary?	Very disagree	0 (0.0)	0 (0.0)	0 (0.0)	0.438	0 (0.0)	0 (0.0)	0.709	0 (0.0)	0 (0.0)	0.010
	Very agree	127 (44.7)	17 (48.6)	110 (44.2)		83 (44.1)	44 (45.8)		61 (55.0)	66 (38.2)	
	disagree	1 (0.4)	0 (0.0)	1 (0.4)		1 (0.5)	0 (0.0)		0 (0.0)	1 (0.6)	
	not sure	17 (6.0)	0 (0.0)	17 (6.8)		13 (6.9)	4 (4.2)		2 (1.8)	15 (8.7)	
	agree	139 (48.9)	18 (51.4)	121 (48.6)		91 (48.4)	48 (50.0)		48 (43.2)	91 (52.6)	
13-Is the full blood count of hematocrit, the number of platelets, white blood cells (at least every 48 h) in the patient suspected of dengue necessary?	Very disagree	0 (0.0)	0 (0.0)	0 (0.0)	0.575	0 (0.0)	0 (0.0)	0.961	0 (0.0)	0 (0.0)	0.022
	Very agree	123 (43.3)	14 (40.0)	109 (43.8)		80 (42.6)	43 (44.8)		58 (52.3)	65 (37.6)	
	disagree	5 (1.8)	0 (0.0)	5 (2.0)		3 (1.6)	2 (2.1)		1 (0.9)	4 (2.3)	
	not sure	35 (12.3)	3 (8.6)	32 (12.9)		23 (12.2)	12 (12.5)		7 (6.3)	28 (16.2)	
	agree	121 (42.6)	18 (51.4)	103 (41.4)		82 (43.6)	39 (40.6)		45 (40.5)	76 (43.9)	
14-If in a suspected dengue patient, access to full blood count is not possible, should fluid therapy of patient be done?	Very disagree	0 (0.0)	0 (0.0)	0 (0.0)	0.859	0 (0.0)	0 (0.0)	0.851	0 (0.0)	0 (0.0)	0.009
	Very agree	112 (39.4)	14 (40.0)	98 (39.4)		78 (41.5)	34 (35.4)		59 (53.2)	53 (30.6)	
	disagree	0 (0.0)	0 (0.0)	0 (0.0)		0 (0.0)	0 (0.0)		0 (0.0)	0 (0.0)	
	not sure	41 (14.4)	4 (11.4)	37 (14.9)		27 (14.4)	14 (14.6)		4 (3.6)	37 (21.4)	
	agree	131 (46.1)	17 (48.6)	114 (45.8)		83 (44.1)	48 (50.0)		48 (43.2)	83 (48.0)	

Eighty-one percent of respondents had a positive attitude about the fact that tires, containers, and pots around the houses are suitable places for the development of dengue vectors, which was statistically significant between men and women (*p* = 0.002). Sixty-two percent of the respondents believed that the government alone is not responsible for controlling dengue fever, and people should be actively involved (93.7%), which was statistically significant between physicians and health experts, while 26.8% considered that the government is solely responsible. Fifty-one percent of the respondents strongly agreed and agreed that PCR and ELISA techniques are used to confirm dengue and 38% were not sure. Seventy-six percent believed that dengue is treatable, with a significant difference in gender (*p* = 0.003), and its reporting should be a national priority (85.3%). Most respondents (93.6%) believed that follow-up of a patient with suspected dengue is a necessity and that a complete blood count should be done at least every 48 h (85.9%), and if this was not possible, fluid therapy should be initiated in a patient with warning signs (85.5%), which was statistically significant between physicians and health experts (*p* = 0.05). There was no significant difference in the attitude of respondents in the public and private sectors regarding ELISA technique (Table 3).

### Practice on dengue fever

The result of dengue-related practice is shown in Table 4. Seventy-three percent of the respondents knew what control measures would be appropriate in the scenario where dengue vectors are not yet establishment in the country. On the other hand, almost half of the respondents, of whom 53.7% were female, with a *value of p* of 0.03, answered correctly regarding control measures during local dengue transmission.

**Table 4:** Practice towards dengue fever (DF) among healthcare workers in Mazandaran Province, northern Iran, 2022.

	Answer	Total	Private sector	Public section	Sig	Female	Male	Sig.	Physician	Health experts	Sig.
		*n* (%)	*n* (%)	*n* (%)		*n* (%)	*n* (%)		*n* (%)	*n* (%)	
1- What measures should be taken if there is no dengue vector in the country?	Correct *	210 (73.9)	28 (80.0)	182 (73.1)	0.285	134 (71.3)	76 (79.2)	0.098	84 (75.7)	126 (76.8)	0.345
	Incorrect**	74 (26.1)	7 (20.0)	67 (26.9)		54 (28.7)	20 (20.8)		27 (24.3)	47 (27.2)	
2-In the case of local transmission of dengue, which operation is used?	Correct***	141 (49.6)	17 (48.6)	124 (49.8)	0.518	101 (53.7)	40 (41.7)	0.036	55 (49.5)	86 (49.7)	0.538
	Incorrect****	143 (50.4)	18 (51.4)	125 (50.2)		87 (46.3)	56 (58.3)		56 (50.5)	87 (50.3)	

*survey of larvae, installation of ovitraps at the points of entry, entomological monitoring throughout the country, human surveillance.

** Immediate reporting of suspected dengue cases.

***Fogging with insecticide, control larvae with insecticide, community engagement for source reduction of the vectors, use of repellent, removal of small water containers around houses and put a lid on the containers to reduce the vector population.

****Use a mosquito coil, installing window screen, pruning grass around homes, use the fan to reduce the vector population, sleeping under a mosquito net at night.

## Discussion

*Aedes*-borne diseases, especially dengue fever, are considered important health issues in many tropical and subtropical countries due to the alarming increase in the number of infected people, disease burden and geographical spread (25). To adopt health development policies in society, increasing knowledge and practice of healthcare workers as well as the general population for mobilizing community actions in the development, maintenance and improvement of the collective and individual health of the people is very important and needed (26). Therefore, the assessment of KAP regarding dengue and its vectors is a research priority both in knowing the extent and impact of health education programs implemented in the past, and at the same time, to understand the health education and training needs of healthcare workers. As no study has been conducted to assess the knowledge, attitude and practice regarding dengue among any target groups in Iran, KAP of healthcare workers regarding dengue was assessed in the northern Province of Mazandaran where the modeling studies highlighted the high risk of the area for the entry and establishment of the invasive *Aedes* species (27).

The present study showed that most of the respondents had heard about dengue (83.8%), and media followed by academic education were the main sources of information. In a study conducted in several countries including India, Indonesia, Myanmar, the Philippines and Thailand, the vast majority of the respondents (> 90%) had heard of the disease in the media (28). Most respondents in other studies, reported that media had been their main source of information on dengue fever followed by healthcare providers (29, 30). This may indicate the important role of media as well as healthcare workers in providing health education programs to change behavior in the community. Despite the fact that a few workshops and continuing medical education were held in the Mazandaran Province about dengue and its vectors before the present study, only a limited number of respondents mentioned it as their source of information. It could probably be either because the participants in the workshops and continuing education did not pass on the information to the health personnel in a cascade education, or the information provided in the workshops and continuing education did not fully cover the relevant objectives. In accordance with our findings, systematic reviews have also highlighted that the effects of continuing medical education on professional practice and health care outcomes is variable and usually unsatisfactory (31, 32). Probably, newer forms of continuing medical education, for example, continuing professional development, are necessary in response to the needs of primary health care workers (4). Nevertheless, these gaps should be revised, planned or modified at the level of the country and/or province, which emphasizes the importance of further studies in this direction.

Our study revealed that healthcare workers had higher knowledge associated with the transmission, clinical management, and prevention and control of dengue compared with its symptoms. In accordance with the present study, several pieces of research conducted in Bangladesh, Malaysia, India and Turkey (33), India (30) and Nepal (1) reported poor knowledge about dengue fever symptoms (6). This lack of awareness of symptoms could be due to (1) the wide range of clinical manifestations observed in patients, (2) the focus of past educational campaigns on transmission, clinical management, and prevention and control rather than on symptoms and primary care, (3) and the disease may be easily confused with other common causes of fever, such as influenza, COVID-19, malaria, typhoid, etc. This has consequences of great importance, because referral to clinics and receiving timely medical care may be delayed until the appearance of severe complications (22, 34).

In contrast, many studies also stated that most participants have a good knowledge of dengue fever symptoms and were able to detect high fever, joint pain and headaches as the main symptoms of the disease (29, 35, 36). One reason for the discrepancy between the results of our study and those aforementioned studies might well be that dengue is not yet epidemic in Iran.

Although, there was a gap in knowledge about the symptoms of dengue diseases, healthcare workers had good knowledge about transmission, clinical management, and prevention and control. Most of them were aware of the transmission routes of dengue (72%) and knew that dengue is both rural and urban disease, which is mostly transmitted after dawn and before sunset by *Aedes* invasive species (37). In agreement with the present study, the same results were also found in other studies in Pakistan including in Punjab (83.8%) by Arif et al. (38), in Karachi (86.9%) by Itrat et al. (39) and in Malakand Khyber Pakhtunkhwa (81.2%) by Khana et al. (39). In contrast to our results, a study from Nepal reported that only 19% (1) of the respondents knew *Aedes* mosquitoes transmit Dengue fever (40).

In the present study, the majority of the respondents were aware of clinical management (82.1%), especially regarding the avoidance of aspirin during dengue compared to other studies in Jamaica (29.8%) (22) and Sri Lanka (42%) (24). Our findings on knowledge of prevention and control (69%) are consistent with those of other studies which reported a fairly good level of knowledge (41) but contrary to those that had reported a low level of Knowledge in this regard (42).

In our investigation, the majority of respondents were classified as having a good attitude (81%) according to the cut-off point described in the methodology (above 70%). This shows that most of them understood the risk of dengue in the country and seem to be ready to support and implement the dengue control programs and measures provided in Iran CDC guidelines for the prevention and control of invasive *Aedes* vectors (13). Similarly, in a study in Central Nepal, high attitude was reported among the healthy population of highland and lowland communities (1). In the present study, 62.6% the respondents had a suitable attitude that the government alone is not responsible for dengue control and believed that it is impossible to reduce the prevalence of dengue without community participation (43). In Karachi Pakistan, 61 % of the respondents believed that dengue control should be the responsibility of the government (44). In addition, there was also a low attitude in response to whether ELISA and PCR methods were suitable for dengue confirmation. Therefore, this reinforces the need to improve the level of respondents’ attitudes in these regards.

A significant difference was observed in the respondents’ knowledge about the clinical management of the disease between the physicians and health experts (*p* < 0.05), which is probably due to the fact that the physicians are more familiar with the relevant concepts than the health experts. This is supported by a study by Huang, Chiu (35). In terms of attitude and practice, females have shown a better attitude and practice than males in response to the questions (attitude: Q1,2,3,5,11) and (practice: Q2). It can be attributed to the higher attention and intension of females in receiving educational concepts due to their major role in households including worrying about children getting sick, collecting and storing water for domestic uses, and home environment sanitation in line with the fact that dengue vectors are mainly domestic and peri-domestic breeders (37). In conformity with our findings, gender as a predictor showed that females have better attitudinal and practical behavior than males (*p* < 0.05) (45, 46). In addition, physicians had a better attitude compared with health experts in response to the question (attitude: Q4,6,7,12,13,14), showing that they are more familiar with the relevant concepts than health experts. The attitude towards PCR and ELISA as diagnostic tools in physicians were less than the cut off of 70%, indicating the necessity of more emphasis on these elements in future continuing education.

In the present study, a translation of knowledge and attitude into practice was observed. Seventy-three percent of the respondents stated that a survey of larvae, installation of ovitraps at the points of entry, entomological surveillance across the country and disease surveillance are the most important strategies to prevent dengue when vectors of the disease are absent in the country. In a study conducted in Central Nepal by Dhimal et al., 90% of participants had translated their knowledge and attitude into actual practice (1). However, there was a difference in the translation of knowledge and attitude into practice in response to the question “What are the appropriate control operations in cases of local dengue transmission.” Forty-nine percent of the respondents stated that fogging with insecticide, controlling larvae with insecticide, promoting community participation to reduce vector breeding sites, use of repellents, removal of small water containers around houses and putting a lid on the containers are useful to reduce the population of mosquitoes.

In accordance with our research, less than half of the respondents with good knowledge about dengue had poor preventive practice, indicating that the translation from knowledge to practice was not properly implemented among the respondents (1, 22, 47). These researchers believed that the socioeconomic status of the participants was likely a limiting factor in translating knowledge into practice. Since there are no reports of local transmission of dengue in Iran, it likely had an impact on translating knowledge into practical measures among the respondents of the present study. Therefore, there is a concern that poor practice in some aspects along with high travel and trade, tourism, and suitable environmental conditions for the establishment and distribution of vector species, may put the province at greater risk. In addition, it should be noted that other parts of the country may be at risk of invasion of the dengue vectors, therefore, continuing education followed by KAP studies should be planned and implemented with priority in high risk provinces based on forecasting, entomological and remote sensing studies (27, 48).

As discussed, there were some discrepancies between the results of our study and those in the literature, which could be due to the different methodologies implemented in the studies including a difference in data analyses, scoring systems or cut off points for “poor” and “good” KAP, the focus of questions in the questionnaire and demographic background of the respondents. Also undertaking this study by virtual means during COVID-19 pandemic might have caused some of these discrepancies. Therefore, the comparison of the results of this research and those of the others should be interpreted with caution.

## Conclusion

The present study provides important insights into the knowledge, attitude and practice of healthcare workers regarding dengue in northern Iran where it is considered a potential focus for the entry of invasive *Aedes* species and-related diseases. The results can help health authorities determine the level of KAP in the healthcare workers to be considered in planning for future training programs. This study showed that although the majority of the study population had good general knowledge, attitude and practice toward dengue, there were gaps in the knowledge of symptoms, the attitude of confirmation techniques (ELISA and PCR), and the role of government in control of dengue, and practice on what control measures are appropriate in case of local transmission of dengue in the country. Considering the importance of high levels of KAP in healthcare workers in providing adequate public health services, it is recommended to design and implement various educational interventions with the aim of improving the knowledge, attitude, and their translation into practice in healthcare workers about various dimensions of dengue, especially where there was a gap. Designing and implementation of the COMBI program for behavior change regarding dengue can also be beneficial.

## Data availability statement

The original contributions presented in the study are included in the article/Supplementary material, further inquiries can be directed to the corresponding author.

## Ethics statement

The studies involving human participants were reviewed and approved by Mazandaran University of Medical Sciences (with ethic code (IR.MAZMS.REC.1398.1107). The patients/participants provided their written informed consent to participate in this study.

## Author contributions

AE, MZ, and SHN: conceptualization, visualization, supervision, and project administration. SHN and MF-D: methodology. SHN and MM: formal analysis. SHN: investigation, resources, data curation, and writing—original draft preparation. AE, MZ, and MMS: writing—review and editing. AE and SHN: validation. AE: funding acquisition. All authors contributed to the article and approved the submitted version.

## Funding

This work was supported by the Vice-Chancellor of Research and Technology of Mazandaran University of medical Sciences [6053]. The funder of the study had no role in study design, data collection, data analysis, data interpretation, writing of the manuscript, or the decision to submit for publication.

## Conflict of interest

The authors declare that the research was conducted in the absence of any commercial or financial relationships that could be construed as a potential conflict of interest.

## Publisher’s note

All claims expressed in this article are solely those of the authors and do not necessarily represent those of their affiliated organizations, or those of the publisher, the editors and the reviewers. Any product that may be evaluated in this article, or claim that may be made by its manufacturer, is not guaranteed or endorsed by the publisher.
