# Supplementary material for: Knowledge, attitude, and practice of healthcare workers regarding dengue fever in Mazandaran Province, northern Iran
Source: Front Public Health. 2023 Jul 4;11:1129056. doi: 10.3389/fpubh.2023.1129056 (PMC10352843; doi:10.3389/fpubh.2023.1129056)
Supplement: Supplementary file 1 [file Table_1.DOC]

Appendix A1: Knowledge, Attitudes, and Practices of Dengue Survey – English Version

Instructions: Please place a check mark in the box that best selects your answer choice.

| **Section 1: Demographics** | | |
| --- | --- | --- |
| What is your role in the medical community | - - Health experts | - - Physician   - General   - Specialist |
| Where is your workplace? | - - Private sector | - - Public sector |
| Where is the city where you work? |  | |
| what gender are you? | - - Male | - - Female |
| How old are you? |  | |
| Have you heard about dengue? | - - - Yes | - - - No |
| If yes, what is your source of information? | - Continuous education | - Academic education |
|  | - Workshop | - Media |

| **Section 2. Knowledge** | | |
| --- | --- | --- |
| **Section 2.1: Knowledge about dengue symptoms** | | |
|  | **Yes** | **No** |
| 1-Is fever one of the symptoms of dengue? |  |  |
| 2-Is headache one of the symptoms of dengue? |  |  |
| 3-Is joint pain one of the symptoms of dengue? |  |  |
| 4-Is muscle pain one of the symptoms of dengue? |  |  |
| 5-Is eye behind pain one of the symptoms of dengue? |  |  |
| 6-Is skin rash one of the symptoms of dengue? |  |  |
| 7-Is abdominal pain one of the symptoms of dengue? |  |  |
| 8-Is diarrhea one of the symptoms of dengue? |  |  |
| 9-Is coughing one of the main symptoms of dengue? |  |  |
| 10-Is chest pain one of the main symptoms of dengue? |  |  |
| 11-Is dizziness one of the main symptoms of dengue? |  |  |
| 12-Is microcephaly one of the symptoms of dengue? |  |  |
| 13- Is the swelling of hands and feet and arthritis obvious symptoms of dengue? |  |  |
| 14-Is conjunctivitis an obvious symptom of dengue? |  |  |
| **Section 2.2: Knowledge about dengue transmission** | | |
| 1- Are flies the main vectors of dengue? |  |  |
| 2- Are ticks the main vectors of dengue? |  |  |
| 3- Are *Anopheles* vectors of malaria, vectors of dengue? |  |  |
| 4- Are *Aedes* mosquitoes vectors of dengue? |  |  |
| 5- Does sexual intercourse transmit dengue disease? |  |  |
| 6- Does person-to-person contact transmit dengue disease? |  |  |
| 7- Does blood transfusion transmit dengue disease? |  |  |
| 8- Is dengue an urban disease? |  |  |
| 9- Is dengue an rural disease? |  |  |
| 10- Is dengue an urban-rural disease? |  |  |
| 11- Is the transmission of dengue disease by the vector mainly done during the day? |  |  |
| 12- Is the transmission of dengue disease carried out by the vector mainly both during the day and at night? |  |  |
| **Section 2.3: Knowledge about the clinical management** | | |
| 1- Do you prescribe aspirin for dengue? |  |  |
| 2- Do you prescribe corticosteroids for dengue? |  |  |
| 3- Is dengue a reportable disease? |  |  |
| **Section 2.4: Knowledge about the prevention and control** | | |
| 1- Is there a vaccine to prevent dengue? |  |  |
| 2- Where do vectors of dengue develop? |  |  |
| Man-made containers (buckets, used tires, drinking water storage barrels) |  |  |
| Riversides |  |  |
| Wide reservoirs |  |  |
| Rice fields |  |  |
| Dirty stagnant water |  |  |
| Animal waste |  |  |
| Pastures and stables |  |  |
| 3- Which cases are used in the individual prevention of dengue disease? |  |  |
| Insect repellents |  |  |
| Long loose white shirt and pants |  |  |
| Lace window |  |  |
| Personal hygiene measures |  |  |
| Daily intake of vitamin C |  |  |
| Sleeping under a mosquito net during the night |  |  |
| 4- Which of the following cases is the combat method of dengue disease? |  |  |
| Destroying or emptying containers containing water |  |  |
| Covering household containers for water storage |  |  |
| Use of chemicals (larvicide in containers containing water) |  |  |
| Solid waste management |  |  |
| Continuous cutting of grass |  |  |
| Storing used tires under the roof or keeping them safe from the rain |  |  |
| Water management in rice fields |  |  |

| **Section 3:Attitude** | | | | | |
| --- | --- | --- | --- | --- | --- |
| In your opinion: | Very agree | Agree | Not sure | Disagree | Very disagree |
| 1. In your opinion, dengue is a dangerous disease? |  |  |  |  |  |
| 2-Is Iran at risk of invasive vectors of dengue? |  |  |  |  |  |
| 3-Is the dengue disease preventable? |  |  |  |  |  |
| 4-Is the control against dengue vectors a strategy to prevent disease? |  |  |  |  |  |
| 5-Do you think that tires, dishes and pots around the house are the proper places for the development of dengue vectors? |  |  |  |  |  |
| 6-Do you think people should actively participate in dengue control? |  |  |  |  |  |
| 7- Do you think just the government is responsible for controlling dengue vectors? |  |  |  |  |  |
| 8- Do you think molecular test is used to confirm Dengue? |  |  |  |  |  |
| 9-Do you think ELISA test is used to confirm Dengue's disease? |  |  |  |  |  |
| 10-Do you think the report of dengue is a national priority? |  |  |  |  |  |
| 11-Do you think that dengue disease can be treated? |  |  |  |  |  |
| 12-Is the follow-up of suspected dengue disease necessary? |  |  |  |  |  |
| Is the full blood count of hematocrit, the number of platelets, white blood cells (at least every 48 hours) in the patient suspected of dengue necessary? |  |  |  |  |  |
| 14- If in a suspected dengue patient, access to full blood count is not possible, should fluid therapy of patient be done? |  |  |  |  |  |

| **Section 4: Practice** | | |
| --- | --- | --- |
|  | Yes | No |
| 1- What measures should be taken if there is no dengue vector in the country? |  |  |
| Survey of larvae at points of entry |  |  |
| Installation of ovitrap at entry points |  |  |
| Entomological monitoring throughout the country |  |  |
| Human care |  |  |
| Immediate reporting of suspected dengue cases |  |  |
| 2-In the case of local transmission of dengue, which operation is used? |  |  |
| Fogging with insecticide |  |  |
| Community engagement for source reduction of the vectors |  |  |
| Control larvae with insecticide |  |  |
| Use a mosquito coil to reduce the vector population |  |  |
| Installing on windows screen to reduce the vector population |  |  |
| Use the fan to reduce the vector population |  |  |
| Sleeping under a mosquito net at night |  |  |
| removal of small water containers around houses to reduce the vector population |  |  |
| Pruning grass around homes to reduce the vector population |  |  |
| Use of repellent |  |  |
| Put a lid on the containers to reduce the vector population |  |  |
